# Sol-Gel Synthesis of Nanocrystalline Mesoporous Li_4_Ti_5_O_12_ Thin-Films as Anodes for Li-Ion Microbatteries

**DOI:** 10.3390/nano10071369

**Published:** 2020-07-14

**Authors:** Jadra Mosa, Mario Aparicio

**Affiliations:** Instituto de Cerámica y Vidrio, CSIC, 28049 Madrid, Spain; maparicio@icv.csic.es

**Keywords:** Sol-Gel, Thin film, Li_4_Ti_5_O_12_, Li-ion microbatteries, Nanocrystalline materials, mesoporous

## Abstract

The development of anodes based on Li_4_Ti_5_O_12_ (LTO) for lithium ion batteries has become very important in recent years on the basis that it allows a long service life (stability in charge-discharge cycling) and safety improvements. The processing of this material in the form of thin film allows for greater control of its characteristics and an improvement of its disadvantages, namely reduced electrical conductivity and low diffusion of lithium ions. In this work, we try to limit these disadvantages through the synthesis of a mesostructured carbon-doped Li_4_Ti_5_O_12_ thin-film with a pure spinel phase using a combination of a block-copolymer template and in situ synthesis of Li-Ti double alkoxide. Structural and electrochemical characterization has been carried out to determine the best conditions (temperature, time, atmosphere) for the thermal treatment of the material to reach a compromise between crystallinity and porosity distribution (pore size, pore volume, and interconnectivity).

## 1. Introduction

The development of miniaturized and flexible and power supply systems in electric cars is a critical point to produce tailor-made microdevices such as nanosensors, smart-cards, and microsystems [1,2,3,4,5,6]. Li-ion battery (LIB) technology is a very promising aspirant for powering such microdevices, due to their fairly high volumetric and gravimetric capacities and energy densities, as well as their large cycling lives and low discharge rates [1,4]. However, the options for reducing cell size in the design of LIBs using a liquid electrolyte are very limited. All-solid-state batteries emerge as a promising alternative due to the use of solid electrolytes that improve safety and chemical stability, also enhancing the electrode–electrolyte interfaces [5,6,7,8]. They also offer an enhancement of Li diffusion kinetics, operation time, and present the opportunity to work at higher temperatures. The latter feature allows for the integration of components onto a solid-state battery in a miniaturized way, and could be useful to generate energy on a large scale [9]. So, the facile scalability of ceramic all-solid-state batteries makes easily transferrable the thin film micro-battery architectures to the portable powered devices industry [10]. Such all-solid-state LIB cells with thicknesses less than 20 µm comprise the development of solid electrolytes and thin film electrodes [1,4,11,12,13,14].

Currently, commercialized graphite, used as commercial anode, does not meet the conditions of safety and rate performance to be applied in battery electric vehicles (EV) and hybrid electric vehicle (HEV)**.** However, lithium titanate, Li_4_Ti_5_O_12_ (LTO), is a promising candidate to replace graphite as an anode material in LIBs, avoiding the formation of solid electrolyte interphase (SEI), losses of Li^+^, and improving LIB performance. Another advantage is the zero-strain during intercalation which imparts LTO with a very long lifespan [15,16,17,18,19,20,21,22]. Nevertheless, LTO has a low theoretical capacity (175 mAh g^−1^) and a low conductivity (10^−8^–10^−13^ Scm^−1^) [23]. To increase conductivity, many efforts have been made, including the achievement of a reduction in particle size [24], nanoporous materials [24,25], carbon coating [26], doping with alkali and alkaline earth metals ions [27,28], and doping with transition metal ions having higher valence than Ti^4+^ [29,30,31,32].

Sol-gel synthesis is an interesting approach to prepare nanostructured materials by controlling their morphology and structure. For example, our previous results describe a mesoporous LiFePO_4_ cathode formed by crystalline nanoparticles arranged in a 3D porous network with a diameter of 20 nm presenting a high surface area with an open connected architecture showing fast Li-ion diffusion and a good interface with the electrolyte [33]. In addition to these advantages, the nanometer scale reduces deformation stress and easily accommodates volume changes that occur during charge–discharge cycles. Soft-templated mesostructured materials using block copolymers such as poly(isobutylene-b-polyethylene oxide) (PBI-b-PEO) were used as a structure directing agent for the assembly of complex oxides [34]. Block copolymers form micelles in the solution thanks to the high hydrophobic/hydrophilic contrast and the self-assembly mechanism, which in this case is called Evaporation Induced Micellar Assembly (EIMA), and an assembly of rigid spheres, surrounded by the inorganic precursor, takes place [34]. The high thermal degradation temperature of the hydrophobic core of the micelle (250–300 °C) allows for the complete dehydration and stabilization of the inorganic phase before decomposition of the organic surfactant, preventing mesoporosity collapsing and retaining carbon in mesostructured walls that act as a rigid support, enhancing electron conductivity.

Few researches have addressed the issue of nanoporous or nanocrystalline Li_4_Ti_5_O_12_ synthesis by soft chemical methods [35,36,37,38,39,40,41,42]. In a former report, we described a sol-gel method to obtain LTO anodes by dipping producing in situ lithium ethoxide that reacts with titanium alkoxide to form a double Li-Ti alkoxide without particle precipitation. This synthetic method gets the spinel phase without the typical impurities (anatase or rutile) thanks to the stoichiometry control at a molecular level that also allows for a reduction in the temperature and time of the thermal treatment (15 min at 700 °C) [22].

In this paper, a novel strategy was proposed for the synthesis of mesoporous nanocrystalline LTO thin-films, combining the advantage of the in-situ Ti-Li double alkoxide prepared by sol-gel and the synthesis of mesoporous nanocrystalline LTO-carbon composites with large and uniform pores of 20 nm via a block-copolymer assembly using PBI-b-PEO. This nanostructure of pores interconnected throughout the material allows very high capacity values that are hardly affected by the increase in current density during charge-discharge cycles.

## 2. Materials and Methods

Titanium isopropoxide (97%, ABCR, Karlsruhe, Germany) and lithium (99.9%, Aldrich, St. Louis, MO, USA) were used as precursors. Absolute ethanol (Panreac) and acetic acid (100%, Merck, Darmstadt, Germany) were used as solvent and hydrolyzing compound, respectively, and poly(isobutylene-b-polyethylene oxide) (P4973-ibEO, noted as PBI-b-PEO, Polymer Sources Inc., Dorval, Canada ) was used as a complexing agent. Absolute ethanol was distilled three times in order to eliminate water residues using a rotary evaporator under the following conditions: refrigeration temperature = 5 °C, pressure = 82 mbar, bath temperature = 46 °C. Then, ethanol was dried with a molecular sieve (0.4 nm—pellets, 1.6 mm diameter from Sigma-Aldrich, St. Louis, MO, USA) for 24 h and then filtered with a 45-micron nylon membrane filter in order to eliminate impurities.

Three types of substrate were used for the coatings, namely quartz, silicon, and gold-coated quartz. Quartz and silicon substrates were used for the structural and chemical characterization of coatings. In order to electrochemically characterize the coatings, quartz substrates covered with a gold layer of 60 nm deposited by a metallization process were used.

For the synthesis of the sol, the molar ratio of Li:Ti:ethanol:acetic acid is 1:1:16:4.8. The procedure was performed in an Ar atmosphere inside a glove box with water content below 5 ppm. The corresponding molar amount of Li was weighed and the metal strips were cut into small fragments (1–1.5 × 0.4–0.6 cm). The metallic Li pieces were cleaned previously with ethanol for a few seconds and then added gradually to the reaction vessel using a bath with ethanol previously cooled at –16 °C. The dissolution was carried out slowly in order to avoid lithium losses, because the reaction is very exothermic. During this reaction, the evolution of hydrogen occurs, and bubbling can be observed due to the formation of lithium ethoxide (Equation (1)).

(1) $${\mathrm{CH}}_{3}{\mathrm{CH}}_{2}{\mathrm{OH}}_{\mathrm{exc}}+\mathrm{Li}\;\to {\mathrm{CH}}_{3}{\mathrm{CH}}_{2}\mathrm{OLi}\;+\frac{1}{2}{\;H}_{2}+{\mathrm{CH}}_{3}{\mathrm{CH}}_{2}\mathrm{OH}$$

After the dissolution of all lithium pieces, the sol is transparent and colorless. Then, the bath was retired, and titanium precursor was incorporated drop by drop and stirring at room temperature one day, with the purpose of generating a mixed lithium and titanium alkoxide. After that, acetic acid was incorporated and left to stir at room temperature for two hours, with the aim to support the development of hydrolysis and condensation sol–gel reactions. A solution of the block copolymer (PBI-b-PEO) in absolute ethanol was prepared (0.050 g of block copolymer in 1.927 mL of ethanol) by stirring for 1h and then, the solution was heated at 70 °C for 5 min to get a total dissolution of the PBI-b-PEO. Finally, this solution was added dropwise over that of the precursors, and left stirring for another 24 h obtaining a stable and transparent sol. The substrates (quartz, gold-coated quartz, and silicon) were dipped in the as-synthesized sol and withdrawn at a speed rate of 15 cm min^−1^ under argon atmosphere (inside the glove box) at a temperature of 70 °C using a Evaporation Induced Micellar Assembly (EIMA) method. The as-produced films were heat treated at 350 °C for 12 h to complete the hydrolysis and condensation reactions of the inorganic species and to form the pores network. Additional thermal treatment (from 500 °C to 700 °C for 15 min) was performed in argon atmosphere in order to crystallize the material and prevent the formation of impurities.

### Analytical Methods

Thin films deposited onto a silicon wafer were characterized by spectroscopic ellipsometry in the range 250–900 nm using an M-2000UTM ellipsometer (J.A. Woollam Co., Lincoln, NE, USA). The incident angles were set to 65°, 70°, and 75° and the acquisition time to 10 s. The data were fitted using the CompleteEASE software (version 5.20, J.A. Woollam Co.) taking into account a Cauchy model. Pore volume was calculated using the Bruggeman theory of effective media from the Cauchy’s dispersion relationships.

Characterization of the coatings also includes analysis of the surface and cross-section of thin films by scanning electron microscopy (HITACHI S-4700 field emission, Krefeld, Germany). Particle size and crystal structure of the samples were also analyzed using a transmission electron microscopy (TEM, JEOL 2100 200 kV high resolution equipped with a Gatan CDD camera, (Akishima, Tokyo, Japan)) by scratching the coating followed by wet-grinding of the scratched material with absolute ethanol, then dropping the solution onto carbon-coated copper grids followed by drying under a UV lamp. Atomic force microscopy measurements were performed on quartz-gold substrates using a Cervantes AFM System (Nanotec Electronica S.L., Madrid, Spain). The crystalline structure of the thin films was analyzed using a Bruker D8 Advance X ray diffractometer (Karlsruhe, Germany). A 2θ survey scan has been performed from 10° to 80° at 1° min^–1^ and a sample interval of 0.05°. The particle size was evaluated using Scherrer formula, eliminating machine broadening and the Cu k-2α peak. FTIR spectra were recorded on a Perkin Elmer Spectrum 100 (Waltham, MA, USA) spectrometer in the range 4000–400 cm^−1^ in transmittance mode with a resolution of 4 cm^−1^ onto coated silicon substrates. Raman study was carried out using a Confocal Micro-Raman (Witec alpha-300R, Ulm, Germany) with laser excitation of 532 nm and a 100× objective lens (NA = 0.9) for coatings deposited on gold-coated quartz substrates. The optical resolution diffraction of the confocal microscope is limited to 200 nm laterally and 500 nm vertically, and Raman spectral resolution of the system is down to 0.02 cm^−1^.

The electrochemical behavior of the films prepared on gold-coated Quartz substrates was carried out in an argon filled three-electrode beaker cell using two Li foils as the counter and reference electrodes, and 1M LiPF_6_/(EC-DEC) as liquid electrolyte. Galvanostatic charge/discharge measurements (Multichannel Potentiostat VMP3 from Biologic, Seyssinet-Pariset, France) were conducted on Li_4_Ti_5_O_12_ films using cutoff voltages at 2.0 and 1.2 V versus Li^+^/Li. Charge/discharge cycling was performed at different current intensities of 7, 14, 28, and 56 µA cm^−2^.

## 3. Results and Discussion

### 3.1. Li_4_Ti_5_O_12_ Thin-Films

No phase separation or precipitates were observed in the final solution, which is transparent and uncolorless. Nanostructured Li_4_Ti_5_O_12_ thin films were prepared onto different substrates via sol–gel chemistry and EIMA dip−coating process using PIB−*b*−PEO block/copolymer as templates. During extraction, evaporation forces the assembly of the block-copolymer with the inorganic precursors forming hybrid inorganic–organic layers onto the substrates. The as-prepared films are transparent and homogeneous in all thermal treatments studied. Thermal treatment promotes the elimination of the block-copolymer, creating a 3D interconnected nanoporous network that retains carbon materials onto LTO walls. Also promotes sol-gel reactions (hydrolysis and condensation) and allows crystallization. The wettability of the sol is adequate to obtain uncolored and homogeneous thin-films on the different substrates used (Supplementary Figure S1).

**Figure 1:**
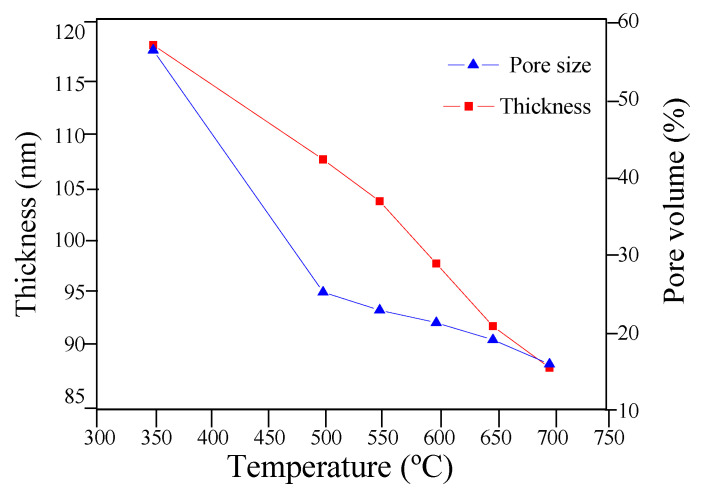
Variation of the thickness and pore volume (%) as a function of the temperature for the mesoporous LTO thin films.

In order to favor the formation of the Li_4_Ti_5_O_12_ structure, a first heat-treatment at 350 C and then a second at various temperatures in the range of 550–700 °C under argon atmosphere for 15 min were performed. Spectral ellipsometry measurements (pore volume and thickness) were performed on silicon substrate at different thermal treatment 350 °C, 500–700 °C (Figure 1). The refractive index of the LTO thin film varies with temperature, was about 1.5 at 350 °C and then increase up to 1.67 at 700 °C (not shown). Also, a decrease of thickness as a function of temperature is observed (120 nm at 350 °C and 90 nm at 700 °C) due the shrinkage of the film after the decomposition of the PBI and PEO groups of the block-*co*-polymer, related to the formation of mesostructured films [43,44]. Figure 1 shows the variation of calculated pore volume as a function of temperature between 350 °C and 700 °C under argon atmosphere. The pore volume decreases from 40 vol.% to 15 vol.% for thin-films heat-treated at 500 and 700 °C, respectively. During heat treatment, contraction of the film in the direction perpendicular to the substrate was produced due to densification and crystallization of the film [33].

### 3.2. Structural Characterization of Li_4_Ti_5_O_12_ Thin-Films

**Figure 2:**
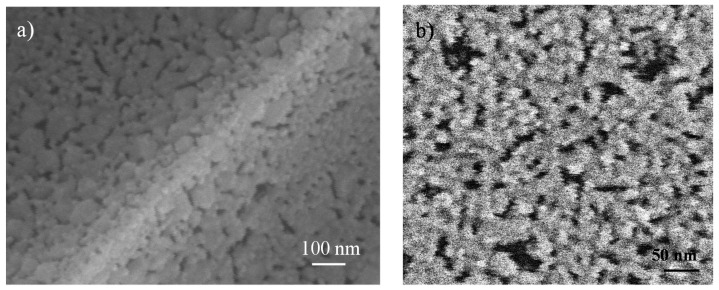
FE-SEM micrographs for L_i4_Ti_5_O_12_ thin-film heat-treated at 700 °C, 15 min (**a**) cross-section and (**b**) surface.

Figure 2a,b shows FE-SEM photographs of cross-section and surface images, respectively, of samples treated at 700 °C for 15 min. LTO thin-film porosity persists after this treatment, although a decreasing of porosity volume due to the crystallization of the matrix is observed. When crystallization, nucleation and growth of the crystals take place, some pores can occlude, deform and even collapse, but the images show that mesostructured still present. A compromise between porosity and crystallization is needed, so the heat treatment parameters, both temperature and time, become key.

**Figure 3:**
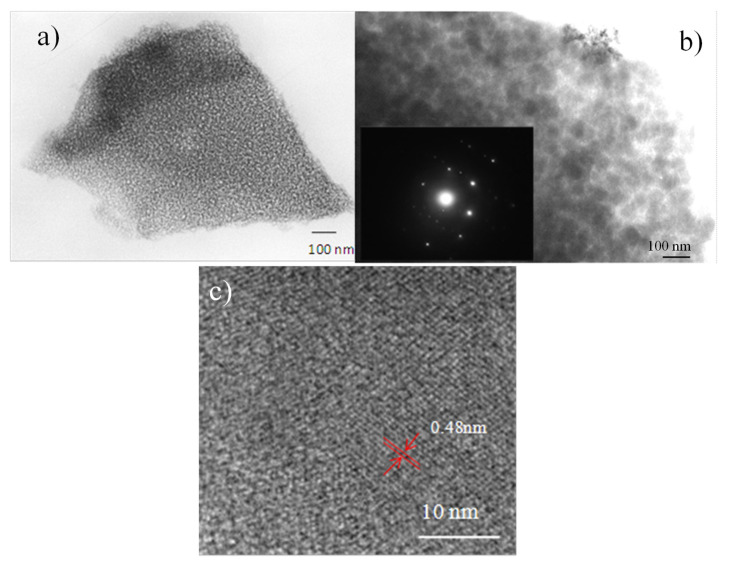
Transmission electron microscopy images and electron diffraction patterns for mesoporous thin films calcined at (**a**) 350 °C, 12 h and (**b**) 700 °C, 15 min with FTT image as inset, and (**c**) TEM image treated with Gwyddion program to index the crystal structure.

Figure 3a shows the HR-TEM micrograph of the LTO thin-films treated at 350 °C for 12 h, showing a typical mesoporous structure with uniform nano-sized particles. Increasing the temperature up to 700 °C (Figure 3b) increases the particle size. The distribution of the particles continues to be homogeneous with sizes of 20–30 nm, maintaining an elevated porosity but losing on some occasions long-range order due to the presence of occluded pores.

Presence of nanocrystalline domains with random orientation in LTO thin-films heat-treated at 700 °C (Figure 3b) were confirmed in HR-TEM images and also in selected area electron diffraction pattern (SAED (inset) that shows Debye–Scherrer rings. Clear lattice fringes with an interplanar distance of 0.48 nm, corresponding to the d-spacing of the (111) crystal plane, are identified (Figure 3c). The calculated lattice spacing is in agreement with JCPDS (Joint Committee on Powder Diffraction Standards) reference card no. 49-0207 for the pure spinel phase Li_4_Ti_5_O_12_ structure [20,22]. Amorphous areas are detected in the images, which can be attributed to carbon content.

**Figure 4:**
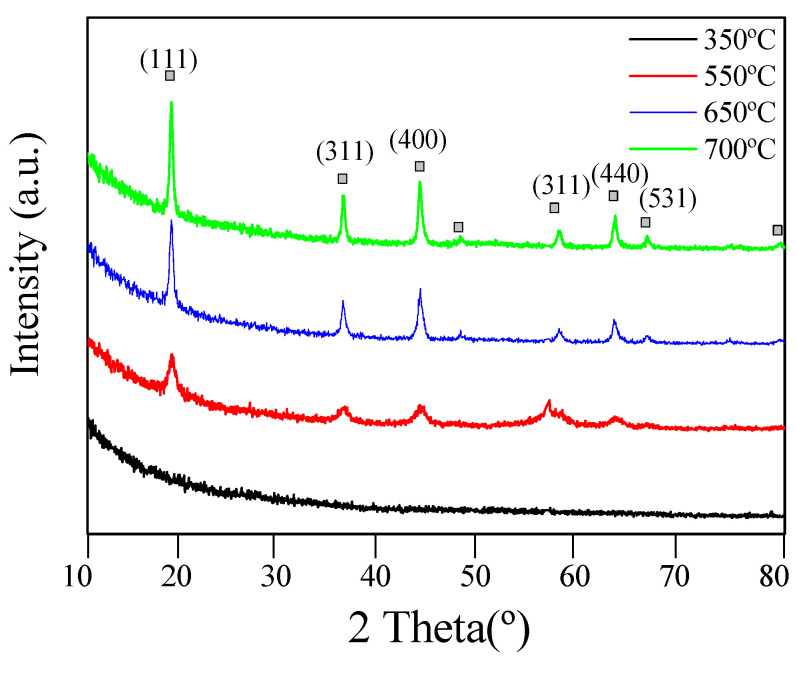
X-ray diffraction patterns of mesoporous Li_4_Ti_5_O_12_ thin films heat-treated at different temperatures under argon atmosphere.

The diffractogram corresponding to the thin-film treated at 350 °C does not show any peak, thus confirming its amorphous nature (Figure 4). Samples heat-treated at temperatures above 550 °C show crystallinity that increases with the sintering temperature. Peaks observed at higher temperatures are narrow and intense, indicating that the crystal structure is well defined. Diffractogram peaks have been indexed confirming that the thin-film presents a pure cubic crystalline structure centered on the face’s spinel (ASTM file: JCPDS # 49-0207), corresponding to Li_4_Ti_5_O_12_ [18,19,21,22]. Presence of impurities or second phases is not observed. These results are in agreement with those obtained by transmission microscopy.

**Figure 5:**
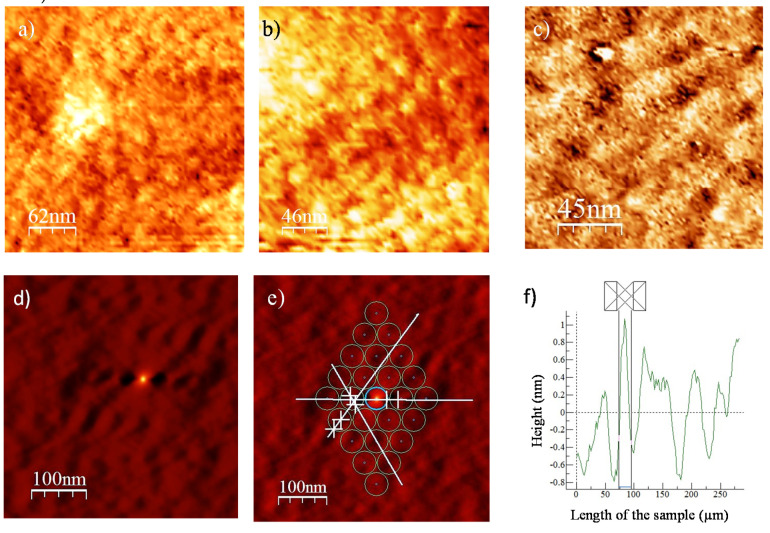
AFM images of mesoporous Li_4_Ti_5_O_12_ thin films calcined at different temperatures: (**a**) 350 °C, (**b**) 550 °C, (**c**) 700 °C; (**d**,**e**) 2D FFT analysis of porosity in 700 °C thin-films in the three directions of cubic arrangement, and (**f**) section graphs in one of the selected direction marked in image (**e**).

Atomic force microscopy (AFM) was used to study Li_4_Ti_5_O_12_ thin-films surface at different thermal treatments and to analyze the periodicity of pores in Li_4_Ti_5_O_12_ thin-film heat-treated at 700 °C (Figure 5).

Figure 5a shows the AFM image of Li_4_Ti_5_O_12_ thin-film heat-treated at 350 °C. The pores are relatively well distributed, homogeneous and mesoporous with an average size of 20 nm. The pore size is fairly comparable to that observed in FE-SEM. Figure 5b,c show the AFM images of surface Li_4_Ti_5_O_12_ thin-film heat treated at 550 °C and 700 °C, respectively. The porous structure of the films keeps at low range order up to 700 °C but open porosity and occluded pores also appeared. 2D FFT was performed also on AFM micrograph of the Li_4_Ti_5_O_12_ thin- film heat treated at 700 °C (Figure 5d,e) and analysis of the image was plotted in Figure 5f. The obtained pattern of five spots equally distant from the center corresponds to cubic symmetry, characteristic of the spinel phase. Pore-to-pore distance in the studied direction was estimated to 45 nm.

**Figure 6:**
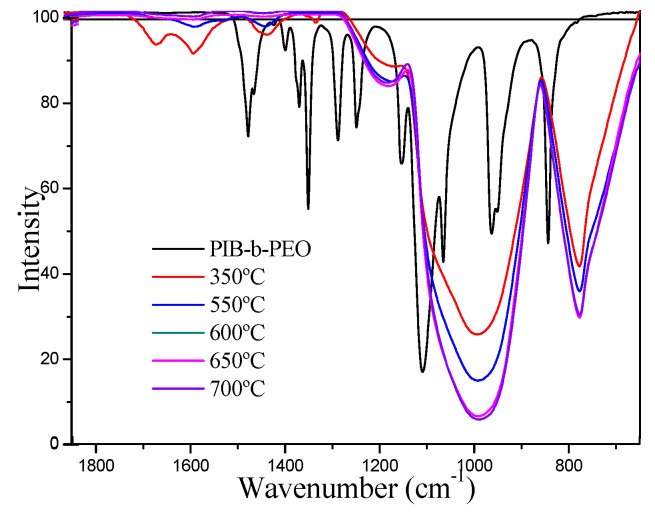
FTIR spectra of mesoporous Li_4_Ti_5_O_12_ thin films heat-treated at different temperatures and PBI-b-PEO block copolymer for comparison.

Figure 6 shows FTIR spectra of the Li_4_Ti_5_O_12_ thin-films heat-treated at different temperatures and also the spectrum of pure PBI-b-PEO block copolymer. Comparing the spectrum of the block copolymer with that of thin films, it can be seen that the main vibration bands disappear. Therefore, FTIR studies confirm that 350 °C intermediate heat treatment is effective because the block copolymer is removed [33]. In the spectra of Li_4_Ti_5_O_12_ thin-films heat-treated at 350 °C and 550 °C with a low degree of sol-gel condensation, a band at 1582 cm^−1^ is observed and assigned to the ν(C = O) stretching vibration mode of CO_2,_ related to presence of lateral chains of the alkoxide and residual reaction products [45]. This band is assigned to the spectra of samples heat-treated at temperatures higher than 550 °C where quite similar and not appreciable changes were observed. The 1450 and 1336 cm^−1^ peaks have been assigned to the strain frequencies of the methylene and methyl bonds [38]. The bands that appear at 1221 and 1062 cm^−1^ are associated with the vibration frequencies of the C–O–C and O–C–C bonds and indicate the presence of esters. At 800 cm^−1^, a band associated with the presence of spinel phase appears [45,46]. At 1000 cm^−1^, a wide band appears that comprises three vibration modes: a stretching vibration band of Si–O–Si as a shoulder at 1100 cm^−1^, a stretching vibration of Li–O–Si at 1040 cm^−1^, and another shoulder around 960 cm^−1^ assigned to Ti–O–Ti frequency mode [47,48,49].

**Figure 7:**
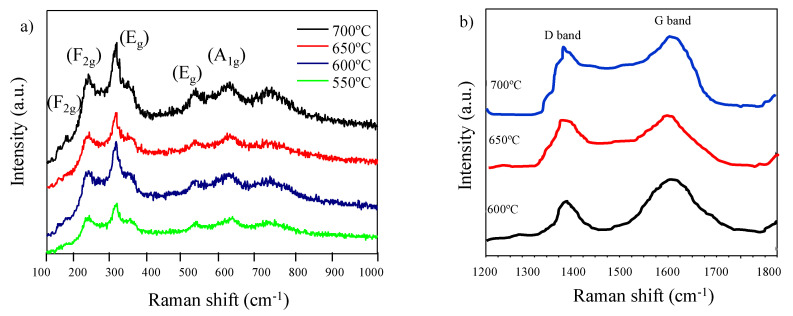
Micro-Raman spectra of mesoporous Li_4_Ti_5_O_12_ thin films heat-treated at different temperatures (**a**) 100–1000 cm^−1^ and (**b**) 1200–1800 cm^−1^.

Likewise, micro-Raman spectroscopy was analyzed on mesoporous Li_5_Ti_4_O_12_ thin- films treated at different temperatures (550–700 °C) to confirm the formation of the perovskite structure (Figure 7a). The spectra show three main bands of vibration at 233, 526, and 654 cm^−1^ associated with the spinel-like structure of the Li_4_Ti_5_O_12_. The main vibration modes of the three possible crystal structures are described in the Table 1.

The absence of anatase and rutile secondary phases of crystallization is confirmed. The spinel-like crystal structure is obtained for all thin-films, which have the vibration modes A1g + Eg + 3F2g. The band at 654 cm^−1^ is associated with the vibration of the Ti–O bonds in the TiO_6_ octahedra (mode A1g). The band at 526 cm^−1^ is related to the stretching vibration of Li-O bonds in LiO_4_ tetrahedra. This band is degenerated with another 334 cm^−1^ band that is not usually observed in the spectra and both correspond to the Eg mode. Finally, the 233 cm^−1^ band is one of the three degenerate bands of the F2g mode and is associated with the vibration of the O–Li–O bonds [40,50,51]. The presence of in-situ formed carbon coming from poly-isobutylene of the PBI-b-PEO block-copolymer is confirmed by the presence of two bands around 1590 cm^−1^ and 1380 cm^−1^ from graphitic carbon (G-band) and disordered carbon (D-band), respectively (Figure 7b) [22]. The D band represents the vibration mode from amorphous carbon and G band originates from graphitized carbon [52,53]. G band presents slightly higher intensity in comparison to D band at all temperatures, suggesting that the graphitized carbon has a slightly higher percentage. A large amount of defects on the surface of graphite grains also contributes to the low peak intensity ratio of the G band to D band [54]. According to HRTEM results, it seems that particles are coated with carbon, and the neighboring particles were connected with an amorphous carbon network. This carbon layer helps to maintain the mesoporous structure during the thermal treatment required to crystallize the perovskite phase, and also enhances the electronic conductivity of the LTO mesoporous anode.

### 3.3. Electrochemical Characterization of Li_4_Ti_5_O_12_ Thin-Films

**Figure 8:**
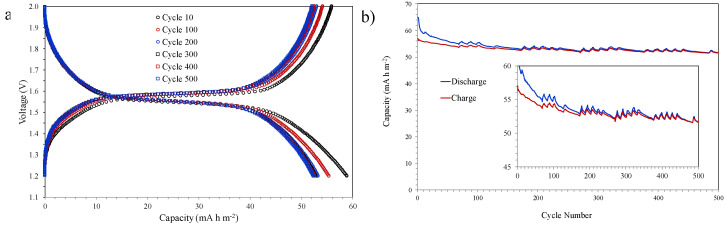
Galvanostatic discharge-charge test at 7 µA cm^−2^ of a sample treated at 600 °C: (**a**) capacities vs. voltage curves at different cycle number, and (**b**) cycle number vs. capacities curves.

The morphological and structural characterization carried out indicates that the increase in the temperature of the thermal treatment leads to an increase in crystallinity and a reduction in thickness and pore volume. The evaluation of LTO coatings as anodes for Li-ion microbatteries has focused on samples treated at higher temperatures (600, 650, and 700 °C) since the formation of the spinel phase at these temperatures has been sufficient and can promote an efficient process of insertion–extraction of lithium ion. Figure 8 shows the results of the galvanostatic discharge–charge test of a sample treated at 600 °C using a current density of 7 µA cm^−2^ and a voltage between 2.0 and 1.2 V (vs. Li^+^/Li).

The capacities vs. voltage curves at different cycle number (Figure 8a) do not present a completely flat plateau as is usual for LTO samples with a pure spinel phase. This behaviour is probably associated with insufficient crystallization due to the low temperature of the heat treatment (600 °C) and its limited duration (15 min). As the number of cycles increases, the discharge capacity decreases, varying between 59 mA h m^−2^ for the cycle 10 and 52 mA h m^−2^ for the cycle 500, although after cycle 300, the reduction slows down, possibly as a consequence of the structure stabilization for the insertion-extraction process of lithium ions. The charge and discharge capacities vs. cycle number (Figure 8b) also show this behaviour, which is also confirmed by the approach between the discharge and charge curves when the cycles number increases. On the other hand, fluctuations are observed in the charge and discharge capacity values that could be related to the limited crystallinity of the structure due to the mild thermal treatment at 600 °C for 15 min.

**Figure 9:**
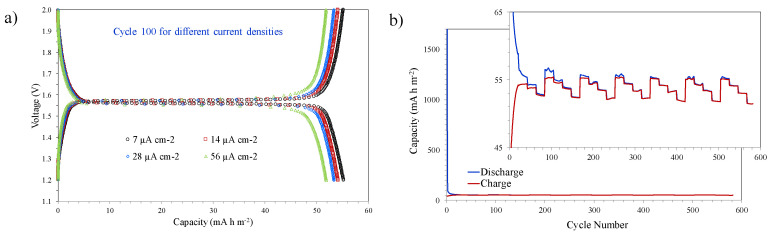
Galvanostatic discharge-charge test at different current densities (7, 14, 28, 56 µA cm^−2^) of a sample treated at 650 °C: (**a**) capacities vs. voltage curves of the cycle 100 for different current densities, and (**b**) cycle number vs. capacities curves with 20 cycles at each current density.

An increase of only 50 °C in the temperature of the heat treatment, maintaining its duration in 15 min, produces significant changes in the electrochemical behaviour. Figure 9 shows the galvanostatic discharge–charge results of a sample treated at 650 °C using different current densities (7, 14, 28, and 56 µA cm^−2^) and a voltage between 2.0 and 1.2 V (vs. Li^+^/Li). The electrochemical behaviour improves substantially with increasing temperature of the thermal treatment, and therefore the representation of the capacity-voltage curves using the same number of cycles would not allow to clearly observing the differences between the samples. The data representation shown highlights these differences in order to analyse the shape of the curves and the flatness of the plateau. In this case, the typical plateau at approximately 1.55 V offered by this type of material is completely flat as a consequence of the increase in crystallinity (Figure 9a). The increase in current density does not seem to significantly influence the flatness of the curve of cycle 100. However, this current increase does cause a slight reduction in capacity values, going from 55 mA h m^−2^ for 7 µA cm^−2^ to 52 mA h m^−2^ for 56 µA cm^−2^, in the case of discharge capacity. This reduction in capacity is very small considering the significant increase in current density, which indicates that the structure (crystallinity, pore connectivity and pore volume) developed with this thermal treatment is very efficient for the insertion-extraction process of lithium ions. Figure 9b shows the charge and discharge capacities vs. cycle number; specifically, groups of 20 cycles have been performed at each current density (from low to high), repeating the 80-cycle period seven times. Some anomalous discharge values during the first cycles are observed, which can be attributed to the interaction of the electrodes with the electrolyte until reaching a state of equilibrium. The results show a stabilization of the electrochemical behaviour as the number of cycles increases, so that the discharge and charge curves come to overlap. A very slight but continuous reduction in capacity values is observed with the number of cycles as a consequence of the structure aging, for example, the value of the discharge capacity at 56 µA cm^−2^ changes from 52.8 mA h m^−2^ at the end of the first group of 80 cycles to 51.5 mA h m^−2^ after the last 80 cycles-group.

**Figure 10:**
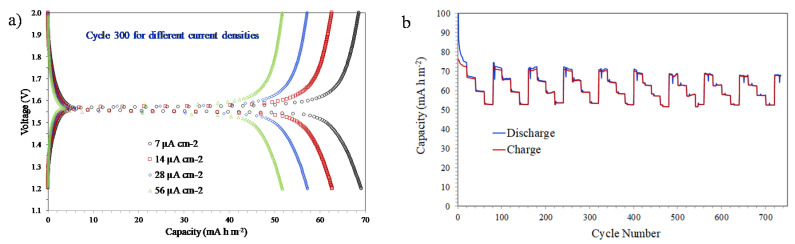
Galvanostatic discharge-charge test at different current densities (7, 14, 28, 56 µA cm^−2^) of a sample treated at 700 °C: (**a**) capacities vs. voltage curves of the cycle 300 for different current densities, and (**b**) cycle number vs. capacities curves with 20 cycles at each current density.

A further 50 °C increase in the thermal treatment temperature also reveals significant changes in the electrochemical behaviour of LTO coatings. Figure 10 shows the galvanostatic discharge-charge results of a sample treated at 700 °C using different current densities (7, 14, 28, and 56 µA cm^−2^) and a voltage between 2.0 and 1.2 V (vs. Li^+^/Li). The typical plateaus at 1.55 V are completely flat, as in the case of the sample treated at 650 °C, indicating that complete crystallization has already been achieved with both thermal treatments (Figure 10a). However, and unlike what is observed in the sample treated at 650 °C, a slightly steeper drop in capacity is observed with increasing current density. This behaviour could be associated with the decrease in pore volume during the thermal treatment at 700 °C that would limit the movement of lithium ions when the current density is higher. The charge and discharge capacities vs. cycle number (Figure 10b) includes groups of 20 cycles performed at each current density (from low to high), repeating the 80-cycle period nine times. As observed in the sample treated at 650 °C, the results show a very stable electrochemical behaviour with hardly any variations in the capacity values throughout the test.

## 4. Conclusions

A simple block-copolymer route in combination with in-situ synthesis of Li–Ti double alkoxide was fruitfully applied to synthesize a nanocrystalline mesoporous carbon-doped Li_4_Ti_5_O_12_ thin-film with a pure spinel structure. Micro-Raman results confirm that part of poly-isobutilene was converted to conductive carbon that helps to maintain the mesostructured porosity during crystallization and also provides electrical conductivity to the Li_4_Ti_5_O_12_ framework. Electrochemical measurements carried out on samples treated at different temperatures indicate that the best results are obtained with the treatment at 650 °C for 15 min, since it combines high crystallinity of the spinel phase and adequate porosity in terms of pore volume and interconnectivity.

## Figures and Tables

**Table 1:** Main assignments of Raman bands of mesoporous Li_4_Ti_5_O_12_ thin-films.

Structure	Vibration Modes (cm^−1^)					
**Spinel**	165 (F_2g_)	233 (F_2g_)	276 (F_2g_)	334 (E_g_)	526 (E_g_)	654 (A_1g_)

## Supplementary Materials

The following are available online at https://www.mdpi.com/2079-4991/10/7/1369/s1, Figure S1: Images of LTO thin-films on (a) quartz, (b) gold-coated quartz and (c) silicon as prepared.
