# Supplementary material for: Sol-Gel Synthesis of Nanocrystalline Mesoporous Li4Ti5O12 Thin-Films as Anodes for Li-Ion Microbatteries
Source: Nanomaterials (Basel). 2020 Jul 14;10(7):1369. doi: 10.3390/nano10071369 (PMC7408516; doi:10.3390/nano10071369)
Supplement: Supplementary file 1 [file nanomaterials-10-01369-s001.pdf]

# Supplementary materials

# Sol-Gel Synthesis of Nanocrystalline Mesoporous $Li_4Ti_5O_{12}$ Thin-Films as Anodes for Li-ion Microbatteries

**Jadra Mosa[1]*, Mario Aparicio[1]**

1 Instituto de Cerámica y Vidrio, CSIC, 28049 Madrid, Spain; jmosa@icv.csic.es (J.M.); maparicio@icv.csic.es (M.A.);

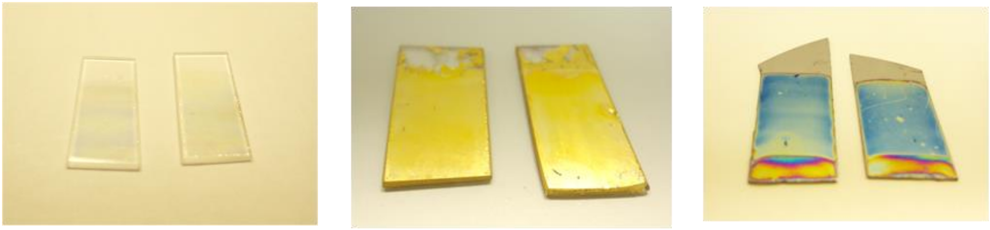

Figure S1. Images of LTO thin-films on a) quartz, b) gold-coated quartz and c) silicon as prepared.
